# Exercise Cardio-Oncology: Exercise as a Potential Therapeutic Modality in the Management of Anthracycline-Induced Cardiotoxicity

**DOI:** 10.3389/fcvm.2021.805735

**Published:** 2022-01-14

**Authors:** Dong-Woo Kang, Rebekah L. Wilson, Cami N. Christopher, Amber J. Normann, Oscar Barnes, Jordan D. Lesansee, Gyuhwan Choi, Christina M. Dieli-Conwright

**Affiliations:** ^1^Division of Population Sciences, Department of Medical Oncology, Dana-Farber Cancer Institute, Boston, MA, United States; ^2^Department of Medicine, Harvard Medical School, Boston, MA, United States; ^3^Department of Epidemiology, School of Public Health, Boston University, Boston, MA, United States; ^4^Department of Health Sciences, Boston University, Boston, MA, United States; ^5^Green Templeton College, University of Oxford, Oxford, United Kingdom; ^6^Department of Environmental and Radiological Health Sciences, Colorado State University, Fort Collins, CO, United States; ^7^Yale University, New Haven, CT, United States

**Keywords:** cardio-oncology, exercise, cardiotoxicity, anthracyclines, cancer survivors, exercise cardio-oncology

## Abstract

Anthracyclines are one of the most effective chemotherapy agents and have revolutionized cancer therapy. However, anthracyclines can induce cardiac injuries through ‘multiple-hits', a series of cardiovascular insults coupled with lifestyle risk factors, which increase the risk of developing short- and long-term cardiac dysfunction and cardiovascular disease that potentially lead to premature mortality following cancer remission. Therefore, the management of anthracycline-induced cardiotoxicity is a serious unmet clinical need. Exercise therapy, as a non-pharmacological intervention, stimulates numerous biochemical and physiologic adaptations, including cardioprotective effects, through the cardiovascular system and cardiac muscles, where exercise has been proposed to be an effective clinical approach that can protect or reverse the cardiotoxicity from anthracyclines. Many preclinical and clinical trials demonstrate the potential impacts of exercise on cardiotoxicity; however, the underlying mechanisms as well as how to implement exercise in clinical settings to improve or protect against long-term cardiovascular disease outcomes are not clearly defined. In this review, we summarize the current evidence in the field of “exercise cardio-oncology” and emphasize the utilization of exercise to prevent and manage anthracycline-induced cardiotoxicities across high-risk and vulnerable populations diagnosed with cancer.

## Introduction

Developed in the 1960s, anthracyclines are one of the most effective chemotherapies, including doxorubicin, daunorubicin, epirubicin, and idarubicin (1, 2). In particular, doxorubicin and daunorubicin have been placed on the World Health Organization's model list of essential medicines since 1977 and 1999, respectively (3, 4). Anthracyclines are used across a broad spectrum of cancers and stages including non-Hodgkin's lymphoma, bladder, and breast cancers (5). However, despite the effectiveness of anthracyclines on eliminating cancer cells, they also have a negative effect on healthy cells leading to acute and long-term side effects such as cardiotoxicities (6–8). The definitions of cardiotoxicity may vary; within this review, we used “toxicity of the heart” or a “decrease in left ventricular ejection fraction” (9). Anthracyclines “attack” the cancer cells by penetrating the nuclear DNA and disrupting the protein synthesis, produce reactive oxygen species, and inhibit topoisomerase II to prevent the repair of DNA (10). However, anthracyclines can also attack healthy cardiac cells, causing the development of toxicities and leading to disabling or fatal cardiac events. Cardiotoxicities may present at any time during the cancer care continuum and are categorized into three stages: acute onset (within the first 2 weeks of treatment), early onset (between the first 2 weeks and 1 year), or late onset (>1 year after treatment) (11). Acute cardiotoxicities, such as tachycardia and arrhythmias, are typically reversible, occur in <1% of cases, and are often eliminated upon cessation of treatment (9, 12); consequently, it is the long-term cardiotoxicities that are of greatest concern, which are often not detected until they are clinically present, and therefore, likely irreversible (9). Preventative strategies for development of long-term cardiotoxicities are a critical unmet need.

Many strategies have been utilized by clinicians to reduce the risk of anthracycline-induced cardiotoxicities. These include: (a) the restriction of the cumulative dose of anthracyclines, particularly to those with cardiovascular disease (CVD) risk factors, (b) use of pegylated liposomes to deliver the therapy, (c) concurrent prescription of dexrazoxane, the only Food and Drug Association approved drug to prevent cardiotoxicities in survivors receiving anthracyclines (9, 13), and (d) long-term monitoring throughout cancer remission for changes in cardiac function and health. Such strategies have reduced the incidence of cardiotoxicities, however, the risk of anthracycline-induced cardiotoxicities is still high (up to 48%) (9, 14). Exercise delivered before, during, and/or after anthracycline treatment is emerging within the cardio-oncology field as a promising strategy to reduce the risk of developing cardiotoxicities (7, 15–17). Exercise improves cardiac strength, function, and capacity and as a result is a well-established component of non-cancer cardiology prevention, prehabilitation, and rehabilitation since 1975 at which time the American Heart Association presented its first exercise guidelines for cardiac exercise rehabilitation (18). While the mechanisms, progression, and cause of cardiotoxicities are likely different in survivors receiving anthracyclines compared to non-cancer cardiac-related conditions, much can be learned from the use of exercise within cardiology and applied to cancer survivors with cardiovascular-related symptoms and conditions.

Various tailored exercise prescriptions have been consistently demonstrated as safe and feasible for many different types of cancers and stages (19). Most commonly, exercise interventions for cancer survivors have been shown to improve cardiorespiratory fitness, physical function, quality of life, fatigue, and body composition, with emerging evidence for improved treatment tolerance and effectiveness (20, 21) as well as reduced long-term cardiac events (22–24). “Exercise cardio-oncology,” a term to our knowledge that is inaugurally coined here, is defined as the application of exercise as a non-pharmacological strategy to prevent, manage, and improve cancer- and treatment-induced cardiotoxicities. This is an underdeveloped field with few longitudinal clinical trials examining the effect of long-term interventions as potentially beneficial strategies for cardio-protection in anthracycline-treated cancer survivors. Therefore, in this review, we summarize the mechanisms of anthracycline-induced cardiotoxicities and present the current pre-clinical and clinical exercise cardio-oncology literature to describe and emphasize the utilization of exercise to prevent, improve, and manage anthracycline-induced cardiotoxicities in individuals diagnosed with cancer.

PubMed, Google Scholar, Web of Science, ClinicalTrials.gov, and NIH RePORTER databases were respectively searched for published studies and ongoing trials which were included until September 2021. Search terms included various combinations of: anthracyclines; cardiotoxicities; cardio-oncology; cardiology; exercise. Secondary searches involved reference lists of eligible articles. The key criterion was to identify studies and ongoing trials that examined cancer survivors, as defined by a person with a cancer diagnosis regardless of treatment status, or animals who were receiving or had received anthracyclines and were undertaking a single bout or long-term exercise intervention and were assessing cardiovascular-related outcomes.

## Mechanisms of Anthracycline-Induced Cardiotoxicities

**Figure 1:**
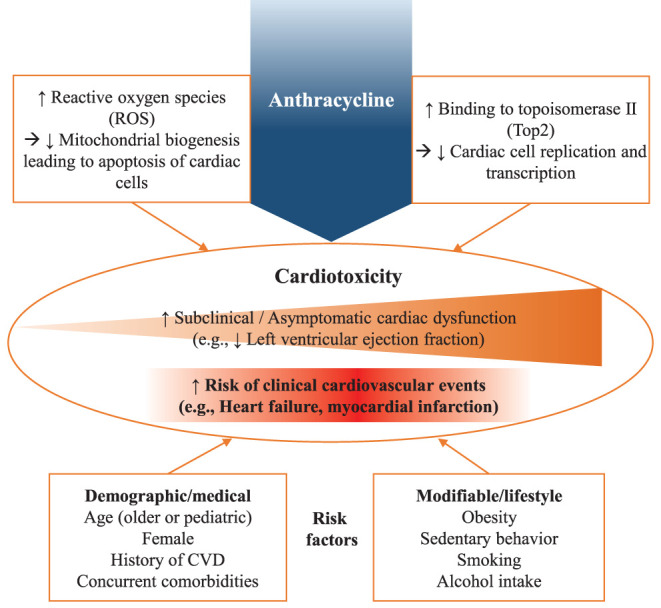
Mechanisms of anthracycline-induced cardiotoxicity.

Anthracyclines are an anti-neoplastic treatment that inhibits the reproduction of cancer cells mainly by blocking DNA synthesis during the cell cycle. Specifically, anthracyclines bind to topoisomerase II (Top2), which plays an important role in adjusting the tension of the DNA pairs for cell replication and transcription (25). Additionally, the increased levels of reactive oxygen species (ROS) induced by anthracyclines can impair mitochondrial biogenesis leading to apoptosis (26). However, the mechanisms of anthracyclines as cancer treatments have been identified as cardiotoxic: Top2 inhibition and ROS accumulation can occur in cardiomyocytes, which impairs mitochondrial biogenesis and results in cardiac dysfunctions such as a decline in left ventricular ejection fraction (LVEF) (Figure 1) (27).

Cardiotoxicities induced by anthracyclines have been extensively reported in epidemiological studies (14, 27). Overall, 6% of survivors who receive anthracyclines experience clinical cardiac events within ~10 years of treatment completion (28); however, the incidence rates can substantially vary depending on cumulative treatment doses, ranging from 3% up to 48% with 400 mg/m^2^ and 700 mg/m^2^ of doxorubicin (DOX), respectively (29–31). Furthermore, the incidence rates increase to 65% when including asymptomatic or subclinical cardiovascular events such as left ventricular dysfunction (LVD) (32). In addition, subsets of survivors who receive anthracyclines are more vulnerable due to several known risk factors that substantially increase the chance of developing CVD (14). They include female sex, older age (>65 years) or pediatric population (<4 years), prior or concomitant chest radiation, concomitant chemotherapy (e.g., trastuzumab), pre-existing cardiac conditions (e.g., hypertension), and other comorbidities (e.g., diabetes and hypercholesterolemia) (6, 33). Lifestyle risk factors are also important contributors to cardiotoxicity such as smoking, high alcohol consumption, obesity, and sedentary behavior (6, 33). Notably, pediatric survivors treated with anthracyclines are at exceedingly high risk and have been shown to have an 8.2 times higher risk of cardiac-related death when compared to their siblings (34).

Subclinical cardiotoxicity, such as LVD, has been an emerging interest in cardio-oncology due to its potential to induce long-term cardiac events. While the definitions for LVD are varied (35), the American Society of Echocardiography and the European Association of Cardiovascular Imaging define it as a reduction in LVEF greater than 10% up to 53% (36). The anthracycline-induced decline in LVEF is classified by the severity of the symptoms (e.g., <53% of LVEF as an abnormality cutoff) and time of appearance (e.g., acute onset (<2 weeks), early-onset (<1 year), or late-onset (>1 year); it is also possible that any onset of cardiotoxicity is a result of a gradual decline of the same disease since the time of first chemotherapeutic treatment (11). Nonetheless, there is consensus that such cardiotoxicities may manifest as early as one week after treatment initiation, although this tends to occur in only 1% of survivors and is typically reversible, or as late as several decades after, in which case the damage in cardiac cells could be irreversible (6, 35). Most anthracycline-induced cardiotoxicities present themselves within 1 year from treatment and are associated with 50% of non-cancer-related mortalities (35). In childhood cancer survivors, the development of clinical cardiac events occurs several decades post-treatment with increasing risks overtime; however, while subclinical disorders such as a reduction in LVEF are common among survivors of childhood cancer, the presenting symptoms are often not detected early enough and lead to clinical CVD (8, 37).

While the mechanisms underlying the toxic effect of anthracyclines in the heart are not yet fully understood, the plausible explanations include that anthracyclines cause injury to healthy cells including cardiomyocytes (6, 27, 35), which constitute 80% of heart mass (38), along with cells providing support to myocytes such as cardiac progenitor cells, cardiac fibroblasts, and endothelial cells (38, 39). The subsequent death of these healthy cells induces cardiomyopathy that can lead to heart failure with symptoms requiring clinical care or, at best, asymptomatic systolic dysfunction indicated by a decline of LVEF (6, 27). Specifically, the generation of ROS by metabolism of anthracyclines that increases cardiac oxidative stress to directly cause damage to DNA, as well as the induction of DNA-strands cleavage by the formation of Top 2β-DNA-anthracycline complex and the suppression of mitochondrial biogenesis have been suggested to induce anthracycline-induced cardiomyopathy (30, 40–42).

Of importance, the series of cardiovascular insults by anthracyclines are also coupled with multiple risk factors including demographic and medical profiles as well as modifiable lifestyle factors, which is labeled as the “multiple-hit” hypothesis by Jones et al. (43). This hypothesis highlights the complexities of cardiotoxicities that not only include the direct cardiac damages of anthracyclines but also the indirect factors that can exacerbate cardiotoxicities such as the systemic effects of treatments on the overall cardiovascular system as well as survivors' with pre-existing or elevated risks of CVD (43). While various pharmacological and monitoring approaches have been employed to prevent or reverse cardiotoxicities in research and clinical settings, there is a lack of non-pharmacological comprehensive interventions that can effectively prevent, improve, or manage cardiotoxicities. Exercise has thereby been proposed as a therapeutic modality that can address anthracycline-induced cardiotoxicities through directly intervening in the biological mechanisms in cardiac cells and cardiovascular system as well as managing pre-existing medical conditions or modifiable CVD risk factors that can worsen cardiotoxicities (44, 45).

## Exercise as Medicine to Improve Anthracycline-Induced Cardiotoxicities

In order to examine exercise cardio-oncology literature among survivors prescribed anthracyclines, a discussion of the current exercise guidelines is required (Table 1). In 2009, the American College of Sports Medicine (ACSM) held the Roundtable on Exercise Guidelines for Cancer Survivors (46), providing an initial framework for the use of exercise in cancer prevention and management. In 2019, a subsequent meeting reflected the exponential rise in interest in the benefits of exercise in oncology, and drew attention to the underlying mechanisms (47). The ACSM and the American Cancer Society (ACS) recommend that cancer survivors should avoid inactivity and undertake regular exercise with the general aim to progress to an accumulation of 150 min of moderate-intensity, or 75 min of vigorous-intensity, per week of aerobic exercise and two strength training sessions, though exercise must be individually tailored to the survivor (47); said guidelines are additionally supported by the American College of Cardiology. Whilst there is strong evidence that exercise improves psychosocial and physical function outcomes, evidence for preventing, managing, and improving cancer- and treatment-related cardiotoxicities is insufficient leading to national recognition of the need for what we have coined here for the first time, exercise cardio-oncology.

**Table 1:** Comparison of the exercise guidelines recommended by the American College of Sports Medicine (ACSM)/American Cancer Society (ACS), American Heart Association (AHA), and American College of Cardiology (ACC).

**Exercise guideline components**	**ACSM/ACS**	**AHA (CORE Guidelines)**	**ACC**
*Frequency and duration*			
Aerobic ≥150 minutes/week over 3–5 days/week	✓	✓	✓
Strength ≥ 2 sessions/week	✓	✓	✓
*Intensity*			
Aerobic-moderate (50–70% peak HR)	✓	Variable	Variable
Resistance (60–70% 1-RM)	✓	Variable	Variable
*Type*			
Aerobic	✓	✓	✓
Resistance	✓	✓	✓
Multi-modality	✓	✓	✓
*Setting*			
Institutional/clinic-based	Nor reported	✓	✓
Home-based	Nor reported	✓	✓
Community-based	Nor reported	✓	✓
*Timing*			
Pre-treatment (diagnosis)	✓	✓	✓
During treatment	✓	✓	✓
Survivorship	✓	✓	✓

*CORE, cardio-oncology rehabilitation; HR, heart rate; RM, repetition maximum*.

In 2019, the American Heart Association (AHA) proposed Cardio-Oncology Rehabilitation (CORE), a multidisciplinary approach to the cardiovascular rehabilitation of cancer survivors based on the well-established cardiac rehabilitation programs for non-cancer cardiology patients (48). CORE integrates structured exercise training with a comprehensive infrastructure of nutritional counseling, weight and blood pressure control, diabetes management, assistance with smoking cessation, and psychosocial support. Specifically, CORE recommends individualized aerobic and resistance-based exercise, based on the aforementioned guidelines from ACSM, for survivors with cancer who are identified as being at increased risk of cardiotoxicity development e.g., those receiving anthracyclines. The implementation of exercise cardio-oncology programs should be considered at all stages of the cancer care continuum e.g., pre, during, and post-treatment, and not only target survivors exposed to high risk cancer-related therapies e.g., anthracyclines, which is the focus of the present review, but also survivors with pre-existing CVD risk factors such as obesity and smoking (32). For example, targeted aerobic and resistance exercise therapy for overweight/obese survivors with early-stage breast cancer can significantly reduce their Framingham risk score and therefore reduce their risk of cardiotoxicity development (49). Based on the evolution of these approaches in support of exercise, the subsequent sections acknowledge the probable mechanisms of action exercise elicits on anthracycline-induced cardiotoxicities.

### Mechanistic Actions of Exercise

**Figure 2:**
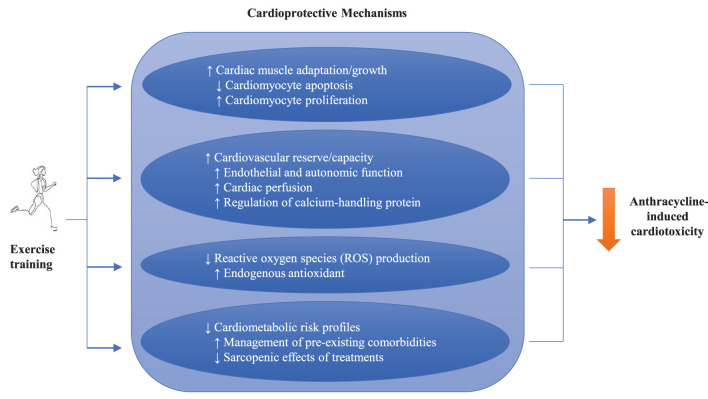
Potential mechanisms of exercise training on anthracycline-induced cardiotoxicities.

The cardioprotective mechanisms of exercise are illustrated in Figure 2. Cardiac muscle does not act in isolation but functions alongside the lungs, diaphragm, peripheral vasculature, nervous system, and metabolism. By improving the function of these other systems, exercise can decrease strain on the heart (50). For example, exercise training may have direct effects on the improvements of cardiac muscle adaptation and growth through enhancing cardiomyocyte proliferation (50). Also, exercise has been shown to decrease oxidative stress or ROS (51) and improve the cardiometabolic risk profiles, in part by challenging the sarcopenic effects of cancer treatments (52). Specifically, exercise has been shown to increase cardiovascular reserve (50, 53, 54), such as by increasing peak oxygen consumption (VO_2peak_) through improved endothelial and autonomic function (55), as well as improved cardiac perfusion (56). Importantly, exercise has been shown to counteract the fall in VO_2peak_ that typically occurs with anthracycline treatment (57). Targeted exercise may also increase cardiovascular reserve by training the diaphragm and decreasing the blood pressure against which the cardiac muscle pumps (58, 59), and additionally has been shown to normalize calcium-handling proteins in cardiac rehabilitation for heart failure (60).

As our understanding grows of the mechanisms by which anthracyclines damage the heart (6, 27, 61), we are able to decipher how exercise can protect myocardial tissue. Owing to the nascence of the exercise cardio-oncology field and the need for non-invasive testing, the mechanistic evidence from human studies is sparse. Nevertheless, several studies have suggested that exercise modulates key biomarkers of cardiac function. Costello, Roberts (62) used cardiac magnetic resonance to show a non-significant but meaningful decrease in longitudinal strain in exercised patients during anthracyclines decreased compared to anthracyclines alone. Also, another study demonstrated that vigorous exercise prior to each DOX treatment can attenuate adverse changes in hemodynamic metrics including cardiac output, resting heart rate (HR) and systemic vascular resistance (63). On a cellular level, the mechanisms by which exercise may protect the myocardium have been studied in murine models. These studies found attenuation of the anthracycline-mediated decreases in end-systolic pressure, left ventricular developed pressure, and maximal rate of left ventricular pressure development (15, 64, 65), through several biomechanisms such as protection against ROS (66–69) by enhancing endogenous antioxidants (70, 71) and heat shock protein action (66, 69).

The cardioprotective mechanisms of exercise training have recently been further investigated by two systematic reviews and meta-analyses. Ghignatti, Nogueira (72) reported a review of 14 preclinical studies and found that trained DOX-treated animals showed significantly better fractional shortening compared to control, with greater benefits from exercise before DOX compared to during or after DOX. The authors highlighted that the most commonly reported cellular mechanism was an exercise-mediated decrease in DOX accumulation in the heart, in addition to the preservation of myosin heavy chain. Similarly, another review by Naaktgeboren, Binyam (73) reported that there is coherent evidence that forced exercise interventions in animals can mitigate DOX-induced cardiotoxicities, where the most commonly reported mechanism of cardioprotection is increased antioxidant production, followed by the enhanced activity of heat shock proteins and the regulation of apoptosis. Other mechanisms include the improved turnover of myocardial tissue by regulating apoptosis (74–76) and increasing the formation of cardiomyocyte progenitors (77), modulation of autophagy and lysosomal signaling (78), and normalization of myocardial calcium activity (79, 80). Although caution must be taken with interpretation because some preclinical models incorporate exercise frequencies and intensities that would not be feasible in humans (81), exercise poses a potential non-pharmacological strategy to protect or even reverse anthracycline-induced cardiac damage in clinical settings.

## Current Evidence in Exercise Cardio-Oncology

**Figure 3:**
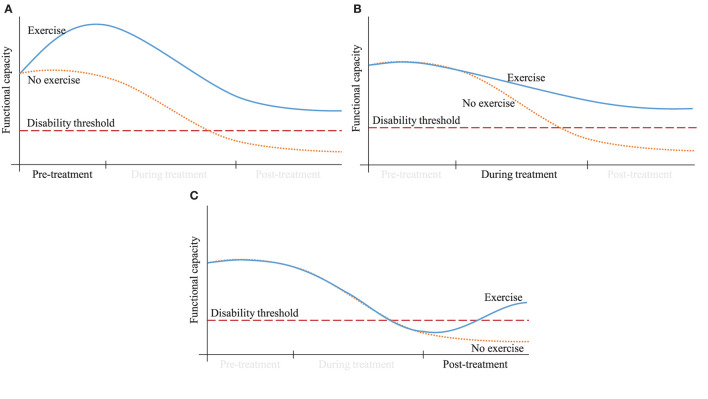
Theoretical representation of how an exercise strategy implemented either at **(A)** pre, **(B)** during, or **(C)** post-treatment could improve a survivor's functional capacity.

Mechanistically, exercise is a viable non-pharmacological strategy that could be implemented at various stages of the cancer care continuum to prevent, manage, and improve anthracycline-induced cardiotoxicities, in addition to other detrimental treatment- and cancer-related side effects such as fatigue, reduced physical function, and altered body composition. Figure 3 provides a theoretical representation of the potential effect of implementing an exercise intervention before, during, or after receiving anthracyclines. Initiated prior to or during anthracycline treatment, exercise may elevate or preserve a survivor's starting point, providing a buffer margin in response to the inevitable treatment-induced decline and preventing a cross into the “disability threshold.” Additionally, exercise implemented after treatment may bring the survivor out of the “disability threshold.” Here we critically evaluate the existing knowledge to understand the impact of exercise implemented before, during, and after anthracycline treatment, along with identifying specific exercise modalities, and their various combinations, that mitigate cardiotoxic-related side effects. We categorized the evidence into intervention modalities (e.g., aerobic, resistance, and multi-modal) and study settings (e.g., preclinical and clinical) to present the full range of adaptations that result from exercise (Table 2).

**Table 2:** Summary of exercise cardio-oncology trials.

**Author, year**	**Study design**	**Primary outcome**	**Subject**	**Treatment**	**Intervention groups**	**Intervention**	**Intervention adherence**	Significant^*^cardiovascular-related outcomes
**Pre-clinical studies**								
***Aerobic exercise***								
Sequeira et al. (82)	RCT	Cardiomyocyte ultrastructure: protein synthesis and oxidative stress reductions	Non-tumor bearing mice	On and post-treatment 1mg/kg DOX hydrochloride 1x/day for 10 days	DOX + exercise (*N =* 16); DOX + sedentary (*N =* 16); Saline + sedentary (*N =* 16)	Aerobic exercise 9 weeks 5x/week 30min/day Treadmill running 50–60% maximal velocity	Not reported	Cardiomyocyte volume density^B^ LV oxidative damage^B^ Superoxide dismutase^B^
Wang et al. (83)	RCT	Ejection fraction and fractional shortening	Model 1: Juvenile tumor bearing mice	Model 1: Pre-treatment 10mg/kg DOX one dose	DOX + exercise (*N =* 8); DOX + sedentary (*N =* 8)	Model 1: 10 days 5x/week 45 min/day Treadmill walking 12 m/min 0% slo	Not reported	DOX in heart tissue^B^
			Model 2: Juvenile tumor and non-tumor bearing mice	Model 2: On-treatment 2.5 mg/kg DOX 2x/week for 2 weeks	DOX + exercise (*N =* 8); DOX + sedentary (*N =* 8); Exercise (*N =* 8); Sedentary: (*N =* 8)	Model 2: 2 weeks 5x/week 45 min/day Other parameters as above.		Fractional shortening^B^ Ejection fraction^B^
			Model 3: Juvenile non-tumor bearing mice	Model 3: Post-treatment Cumulative dose of 25 mg/kg DOX over 5 weeks	DOX + exercise (*N =* 8); DOX + sedentary (*N =* 8); Exercise (*N =* 8); Sedentary: (*N =* 8)	Model 3: 8 weeks 3x/week Other parameters as above.		Not reported
Ahmadian and Roshan, (69)	RCT	Cardiotoxicity	Non-tumor bearing mice	Pre-treatment 20 mg/kg DOX hydrochloride one dose	DOX + exercise (*N =* 8); Doxorubicin (*N =* 8); Saline + exercise (*N =* 8)	3 weeks 5x/week Treadmill running Progressive increase each week 15 to 17 m/min 25 to 39 min	Not reported	Superoxide dismutase^B^ C-reactive protein^B^ Heat shock protein^B^ Malondialdehyde^B^
Hayward et al. (84)	RCT	Cardiac function	Non-tumor bearing mice (juvenile)	On treatment 2 mg/kg DOX 1x/day for 7 days	DOX + sedentary (*N =* 22); DOX + exercise (*N =* 22); Saline + sedentary (*N =* 10); Saline + exercise (*N =* 10)	10-weeks 24-h access to running wheel.	Not reported	HR^B^ Blood velocities^B^ Isovolumetric relaxation time^B^ LV developed pressure^B^ endSP^B^ endDP^B^
**Pre-clinical studies**								
***Aerobic exercise***								
Ashraf and Roshan (67)	RCT	Cardiac oxidative damage biomarkers	Non-tumor bearing mice	Pre-treatment 10 mg/kg or 20 mg/kg DOX hydrochloride one dose	DOX (10 mg/kg) + exercise (*N =* 8); DOX (20mg/kg) + exercise (*N =* 8); DOX (10mg/kg) + sedentary (*N =* 8); DOX (20mg/kg) + sedentary (*N =* 8); Saline + sedentary (*N =* 8); Saline + exercise (*N =* 8)	See Ahmadian and Roshan (69)	Not reported	Malondialdehyde ^B^ Apelin^B^ Superoxide dismutase^B^
Hydock et al. (15)	RCT	MHC and SERCA2a alterations	Non-tumor bearing mice	Pre-treatment 1mg/kg DOX 1x/day for 10 days	DOX + treadmill (*N =* 23); DOX + voluntary wheel running (*N =* 14); DOX+ sedentary (*N =* 17); Saline + treadmill (*N =* 12); Saline + voluntary wheel running (*N =* 17); Saline + sedentary (*N =* 11)	10 weeks 5x/week Treadmill running Or 24-h access Voluntary wheel running	Not reported	SERCA2a^B^ β-isoform MHC^B^ LV mass^B^ Fractional shortening^B^ RWT^B^ SWs^B^ SWd^B^ PWs^B^ PWd^B^
Matsuura et al. (85)	RCT	Platelet L-arginine-nitric oxide pathway and vasodilator properties	Non-tumor bearing mice	Post treatment 1mg/kg DOX 1x/day for 10 days	DOX + exercise (*N =* 12); DOX + sedentary (*N =* 12); Saline + sedentary (*N =* 12); Saline + exercise (*N =* 12)	Aerobic exercise 6 weeks 5x/week 60 min Treadmill running 50–60% maximal velocity	Not reported	Vasodilation of mesenteric vascular bed^B^
***Resistance exercise***								
Feitosa et al. (86)		Cardiac contractility, hemodynamics, baroreflex, cardiac autonomic tonus and oxidative stress	Non-tumor bearing mice	During treatment 2.5 mg/kg DOX 1x/week for 6 weeks	DOX + exercise (*N =* 13); DOX + sedentary (*N =* 13); Control (*N =* 13)	8-week resistance training: 40% 1RM (weighted leg extensions: 3 sets of 10 reps with 60 second rest, 3 × per week)	Not reported	HR^B^ Oxidative stress^B^ Diastolic arterial pressure^B^ LV developed pressure^B^
**Pre-clinical studies**								
***Resistance exercise***								
Pfannenstiel and Hayward (87)	RCT	Cardiac function	Non-tumor bearing mice	Pre-treatment 12.5 mg/kg DOX one dose	DOX + exercise (*N =* 15); DOX + sedentary (*N =* 15); Saline + sedentary (*N =* 9); Saline + exercise (*N =* 9)	Resistance training 12 weeks Bipedal stance (cage lid elevated 1–2.5 cm each week)	Not reported	Fractional shortening^B^ Maximal blood flow through aortic valve^B^ Maximal blood flow through mitral valve^B^ LV developed pressure^B^ MHC isoform distribution^B^
**Clinical studies**								
***Aerobic exercise***								
Lee et al. (88)	RCT	ECM-regulating enzymes: matrix metalloproteinases	Breast cancer (Stage I-III)	On treatment. DOX and cyclophosphamide (every 2 weeks for 4 cycles).	Intervention (*N =* 15); Usual care (*N =* 15)	8 weeks 3x/week 30-min sessions Supervised Cycle-based HIIT 10–90% intervals of PPO	HIIT session attendance was 82.3%.	MMP-9^WI^ MMP-2^WIWC^
Lee et al. (16)	RCT	Vascular endothelial function	See Lee et al. (92)	baFMD ^B, WI, WC^ cIMT ^WC^ Lee et al. (91)	RCT	Cardiorespiratory fitness	See Lee et al. (92)	${\text{VO}}_{2\text{max}}^{\text{WC}}$
Kirkham et al. (63)	RCT	Acute changes in cardiac function	Breast cancer (Stage IIB-IIIC)	Pre-treatment	Intervention (*N =* 13); Usual care: *N =* 11	Acute bout aerobic exercise 70% age-predicted HRR	N/A	NT-pro-BNP^B, WI^ Ejection fraction^WI^ Systolic strain rate^WI^
Hornsby et al. (89)	RCT	Adverse events; cardiopulmonary function, patient-reported outcomes	Breast cancer (Stage IIB-IIIC)	On-treatment Neoadjuvant chemotherapy (4 cycles): 60 mg/m^2^ DOX and 600 mg/m^2^ cyclophosphamide	Intervention (*N =* 10); Usual care (*N =* 10)	12 weeks 3x/week Supervised 15–45 min sessions (progressive design) 60–100% VO_2peak_	Attendance rate was 82%, adherence to protocol was 66%.	Resting HR^WC^ Peak exercise HR^WC^ Oxygen pulse ^B, WI, WC^ ${\text{VO}}_{\text{2peak}}^{\text{B,WI,WC}}$
***Resistance exercise***								
Schmidt et al. (90)	RCT	Fatigue and quality of life	Breast cancer (Stage I-III)	On treatment Adjuvant chemotherapy (89.5% received anthracyclines)	Intervention (*N =* 49); Muscle relaxation control (*N =* 46)	12 weeks 2x/week 60 min Supervised Resistance exercise: Eight machine-based progressive exercises 3 sets, 8-12 repetitions	Median attendance in both groups was 17 out of 24 sessions.	Not reported
**Clinical studies**								
***Multi-modal exercise***								
Ansund et al. (91)	RCT	Long-term myocardial damage and physical capacity	Breast cancer (Stage I-III)	On and post treatment Anthracycline, taxane, or combination of the two.	Resistance + HIIT (*N =* 79); Aerobic + HIIT (*N =* 80); Usual care (*N =* 81)	16 weeks 2x/week Supervised Resistance: 8–12 repetitions at 75–80% of 1RM Aerobic: 3 × 3 min bouts of HIIT at RPE of 16–18 Moderate-intensity and high-intensity aerobic training: 20 min of moderate-intensity at 13–15 on Borg scale followed by 3 × 3 min bouts of HIIT at RPE of 16–18	Not reported	Nt-pro-BNP^B^
Kirkham et al. (92)	Non-RCT	Resting cardiac function and hemodynamics	Breast cancer (Stage I-III)	On treatment 240 mg/m^2^ DOX and 2400 mg/m2 cyclophosphamide over four cycles (2–3 weeks apart).	Intervention (*N =* 26); Usual care (*N =* 11)	Estimated 8–12 weeks 3x/week Supervised (progressions 1–2 weeks) Aerobic 50–75% age predicted HRR Resistance exercise (moderate intensity, whole body)	Median adherence 3x/week: 63%. Adherence to aerobic protocol was 86% for intensity & 96% for duration.	Diastolic strain rate ^WI^ Hemoglobin^WIWC^ Hematocrit^WIWC^ Cardiac output^WC^ Resting HR^WC^ Systemic vascular resistance^B, WI, WC^ Mean arterial pressure^WIWC^ ${\text{VO}}_{2\text{peak}}^{\text{WI}}$
Kirkham et al. (92)	Non-RCT	Resting cardiac function and hemodynamics	Breast cancer (Stage I-III)	On treatment 240 mg/m^2^ DOX and 2400 mg/m2 cyclophosphamide over four cycles (2–3 weeks apart).	Intervention (*N =* 26); Usual care (*N =* 11)	Estimated 8–12 weeks 3x/week Supervised (progressions 1–2 weeks) Aerobic 50–75% age predicted HRR Resistance exercise (moderate intensity, whole body)	Median adherence 3x/week: 63%. Adherence to aerobic protocol was 86% for intensity & 96% for duration.	Diastolic strain rate ^WI^ Hemoglobin^WIWC^ Hematocrit^WIWC^ Cardiac output^WC^ Resting HR^WC^ Systemic vascular resistance^B, WI, WC^ Mean arterial pressure^WIWC^ ${\text{VO}}_{2\text{peak}}^{\text{WI}}$
**Clinical studies**								
***Multi-modal exercise***								
Kirkham et al. (93)	Single group	Cardiovascular autonomic function	Breast cancer (Stage I-IIIA)	On treatment and post treatment. DOX + cyclophosphamide + paclitaxel: *N =* 49 DOX + cyclophosphamide: *N =* 3 Docetaxel + cyclophosphamide: *N =* 21 Anthracycline + another drug: *N =* 73	Intervention (*N =* 73)	Estimated 8–12 weeks 3x/week Supervised Aerobic/Resistance 50–75% HRR/1RM Post treatment 2x/week (week 1–10) 1x/week (week 11–20) Supervised Aerobic intervals 4 × 4 min at 75–85% + 4 min at 40–65% VO_2_/HRR	Attendance ranged from 51–71% depending on treatment plan.	Resting HR^WI^ Resting SBP^WI^ Resting DBP^WI^
Howden et al. (57)	Non-RCT	Cardiovascular fitness	Breast cancer (Stage I-III)	On-treatment DOX, cyclophosphamide (71%) Fluorouracil, epirubicin, cyclophosphamide, docetaxel (11%)	Intervention (*N =* 14); Usual care (*N =* 14)	12 weeks 3x/week 2 supervised sessions per week, 1 home-based session 60 min Aerobic exercise 30min Resistance exercise 30min Periodization plan (2 week loading, 1 week unloading)	Compliance to supervised exercise session: 76%	${\text{VO}}_{2\text{peak}}^{\text{B}}$ Arterio-venous Oxygen difference^B^
Foulkes et al. (94)	Non-RCT	Cardiovascular fitness, cardiac function	Breast cancer (Stage I-III)	Off-treatment 12-months post treatment	Intervention (*N =* 7); Usual care (*N =* 8)	See Howden et al. (57)		${\text{VO}}_{2\text{peak}}^{\text{B},\text{WI},}$ ^WC^
Mijwel et al. (95)	RCT	Cardiorespiratory fitness; Muscle strength			See Ansund et al. (91)			${\text{VO}}_{2\text{peak}}^{\text{B},\text{WI}}$
**Clinical studies**								
***Multi-modal exercise***								
Järvelä et al. (96)	Single group	Myocardial function	Survivors of childhood ALL (>10 years)	Off treatment Cumulative dose of 120–370mg/m^2^	Intervention (*N =* 21)	12 weeks 3–4 × /week Unsupervised home-based Resistance training 8 exercises – as many repetitions as possible for 3 cycles Aerobic exercise 30min (recommended 3 × /week)	Not reported	Early diastolic mitral filling wave (E)^WI^
Smith et al. (97)	Case series	Peak oxygen consumption and exercise tolerance	Survivors of childhood osteosarcoma or Ewing sarcoma (>10 years)	Off treatment	Intervention (*N =* 5)	12 weeks Unsupervised home-based Aerobic exercise 3–5 days/week Resistance exercise 2–3days/week	Compliance to prescribed exercise: 86%	${\text{VO}}_{2\text{peak}}^{\text{WI}}$ Ejection fraction^WI^ Oxygen pulse^WI^
Järvelä et al. (98)	Single group	Cardiorespiratory fitness	Intervention (*N =* 17)	See Järvelä et al., 2016 (96)				${\text{VO}}_{2\text{peak}}^{\text{WI}}$

* *Significance defined as p < 0.05 with the following indicating what was significant: B, between group significance; WI, within group significance for the intervention group; WC, within group significance for the control group. RCT, randomized clinical trial; DOX, doxorubicin; LV, left ventricle; MHC, myosin heavy chain; SERCA2a, sarcoendoplasmic reticulum Ca^2+^ ATPase 2a; RWT, relative wall thickness; SWs, septal wall thickness at systole; SWd, septal wall thickness at diastole; PWs, posterior wall thickness at systole; PWd, posterior wall thickness at diastole; HR, heart rate; endSP, end systolic pressure; endDP, end diastolic pressure; 1RM, one-repetition maximum; HIIT, high intensity interval training; PPO, peak power output; MMP, matrix metalloproteinase; baFMD, brachial artery flow mediated dilation; cIMT, carotid intima media thickness; VO_2maxorpeak_, maximal or peak oxygen consumption; HRR, heart rate reserve; N/A, not applicable; NT-pro-BNP, amino terminal of B-type natriuretic peptide; RPE, rate of perceived exertion; SBP, systolic blood pressure; DBP, diastolic blood pressure; ALL, acute lymphocytic leukemia*.

### Aerobic Exercise

Aerobic exercise is traditionally the most common mode prescribed to improve cardiorespiratory fitness and cardiovascular function, both surrogate measures of CVD risk (99). Aerobic exercise is typically well-received given its familiarity with non-exercisers (e.g., walking), ease to implement in both remote and in-person environments with limited or no equipment required, and includes modes that reduce the impact on the joints (e.g., swimming) (100). Here we discuss the effect of aerobic-based exercise interventions on cardiotoxicities and related cardiovascular outcomes in survivors prescribed anthracyclines.

#### Murine Models

Studies assessing exercise interventions prior to, or concurrent with, anthracycline treatment has been the focus of preclinical trials using aerobic exercise to improve cardiotoxicity outcomes (72, 73). Hydock, Lien (15) examined the effect of a 10-week prehabilitative exercise intervention in preventing the development of DOX-induced cardiotoxicities in rats. Three protocols were compared: treadmill running, voluntary wheel running, and sedentary control, each receiving either saline or DOX. The authors reported that in DOX groups, regardless of modes of activity, exercise was cardioprotective, indicated by the preservation of myosin heavy chain expression and aortic and mitral valve mean and maximal blood flow, which was similar to that in all saline groups and significantly different to the sedentary-DOX control group. Furthermore, the cardioprotective timeline of exercise persisted 4 weeks after completing the 10-day DOX regimen even though the animals remained sedentary during this period. Similarly, studies by Ashraf and Roshan (67) and Ahmadian and Roshan (69) respectively examined cardiovascular-related outcomes in rats using a 3-week aerobic training intervention done prior to anthracycline exposure. Compared to the DOX-sedentary groups, rats in the respective DOX-exercise groups exhibited upregulation in antioxidant markers (e.g., superoxide dismutase, malondialdehyde), further demonstrating the ability for exercise to counteract the increase in oxidative stress of anthracyclines, which has been linked to the development of cardiotoxicities (67, 69). These studies highlight the potential of pre-treatment exercise in preventing DOX-induced cardiotoxicities and the potential in inducing a sustainable effect post-chemotherapy even in an inactive state.

While exercise prior to anthracycline treatment has been shown to have a significant impact on cardiotoxicities, only one study, to date, utilized exercise after treatment and targeted rats with DOX-induced heart failure. Matsuura, Brunini (85) reported survival rate to be significantly improved in those rats with DOX-induced heart failure who undertook 6 weeks of aerobic exercise (67% survived), compared to the DOX-sedentary group (33% survived). Similarly, Sequeira, Martins (82) reported that, the exercised rats during DOX treatment had a 100% survival rate compared to 68.8% in the rats who were sedentary. It should be noted that the above preclinical studies (15, 67, 69, 82, 85) did not utilize rats that were diseased with cancer, as such, the inflammatory and biologic nature of the tumor and how these react with the exercise and anthracycline drug cannot be identified. Wang, Iskra (83) used both tumor and non-tumor bearing juvenile mice and reported: (1) in tumor-bearing mice, 2-weeks of aerobic exercise followed by DOX infusion resulted in less DOX in the heart tissue compared to sedentary controls, (2) in both tumor and non-tumor bearing mice, 2-weeks of aerobic exercise completed concurrently with DOX resulted in preserved fractional shortening and ejection fraction, and (3) in non-tumor bearing mice, 8-weeks of aerobic exercise implemented after DOX resulted in no recovery of fractional shortening or ejection fraction. However, the tumor and non-tumor-bearing juvenile mice were not directly compared, thereby the effect of the tumor is still unclear. While not tumor-bearing, Hayward, Lien (84) also examined a juvenile murine model and reported 10-weeks of voluntary aerobic exercise, performed concurrently with DOX regimen, to preserve HR, blood flow velocities, and isovolumetric relaxation time, all measures of cardiac function, when compared to significant impairments in the DOX-sedentary group. Exercise conducted prior to, or in conjunction with, anthracyclines may be beneficial for pediatric cancer survivors prescribed anthracyclines as demonstrated by the preservation of cardiac-related outcomes in juvenile murine models (83, 84). Overall, the improvement in cardiovascular-related outcomes reported in these preclinical studies offers a promise of aerobic-based exercise as a possible strategy to improve cardiac-related health status and even reduce cardiotoxicity-related mortality, however, further studies with tumor-bearing or human tumor xenograft models are warranted to provide more transferable evidence to human settings.

#### Clinical Interventions

Aerobic-based clinical studies have predominantly focused on exercise during treatment. Hornsby, Douglas (89) implemented a supervised 12-week progressive aerobic-based intervention in breast cancer survivors receiving anthracyclines (*n* = 20) and found that exercise significantly increased VO_2peak_ from 19.5 to 22.1 ml/kg/min when compared to the usual care group who had a statistically significant decline in VO_2peak_ from 17.5 to 16.0 ml/kg/min, which was approaching the cut off value for independence for women at 15 ml/kg/min (101). No changes were observed in any echocardiography measured cardiac function outcomes for either group. Furthermore, the significant improvement in VO_2peak_ occurred irrespective of a reduction of exercise dose by 23% due to nausea, tiredness, or not feeling well, yet exercise session attendance was high (82%). This suggests that the modification of the exercise prescription when survivors are not feeling up to the prescribed program can still result in clinically meaningful benefits, although low and high adherers were not distinguished within the analysis. Supervised exercise during treatment should be encouraged as exercise physiologists will be able to provide appropriate, tailored, real-time advice that will ensure survivor safety within the exercise environment, yet still provide an adequate exercise stimulus to ensure adaptations occur.

Timing of exercise during treatment is a widely debated concept with no clear evidence regarding the most effective programming. However, a randomized controlled trial (RCT) by Kirkham, Eves (63) provides insight into the potential benefits of exercise completed 24 h prior to anthracycline infusion. They examined the effect of a single 30-min treadmill walking exercise session completed 24 h prior to anthracycline infusion on cardiac function in breast cancer survivors (*n* = 27). This acute exercise study reported that a single bout of exercise can attenuate the anthracycline-induced increase in NT-pro-BNP (*p* < 0.05), a marker of cardiotoxic effects, and resulted in an increase in systolic strain rate and LVEF, suggesting exercise to improve systolic function compared to usual care (*p* < 0.05). Implementing a single aerobic bout of exercise prior to each chemotherapy infusion is potentially a feasible and more achievable exercise strategy during treatment than multiple weekly sessions. Further research is required to assess the effectiveness of this timing of exercise when completed for all chemotherapy cycles, and gauge if it provides similar or additional benefits to that seen in weekly exercise sessions throughout treatment.

One promising aerobic exercise modality is high intensity interval training (HIIT), which involves alternating bouts of high (e.g., 90% of peak power output) and low (e.g., 10% of peak power output) intensity movement and is considered an effective form of aerobic training to ameliorate CVD risk factors (102). In a RCT by Lee and colleagues among 30 stage I-III breast cancer survivors during anthracycline treatment (103), an 8-week HIIT intervention significantly improved brachial artery flow-mediated dilation (baFMD) and carotid intima media thickness (cIMT) compared to usual care (16), as well as improved levels of matrix metalloproteinases (MMP) within the HIIT group (88), which are implicated in atherosclerosis development (104, 105). Although these findings provide promising insight into the benefits of HIIT in improving vascular endothelial function (88), the study focused on the feasibility of HIIT during anthracyclines and the cardiovascular outcomes were only exploratory, as such, larger trials with the primary focus on anthracycline-induced cardiotoxicities are needed.

### Resistance Exercise

Traditionally, resistance exercise is prescribed to improve skeletal muscle strength and hypertrophy (106), yet, improvements in skeletal muscle-related outcomes can also lead to the optimization of cardiac function (107). Resistance training is considered a critical component within exercise oncology given its role in the preservation of lean mass which is positively associated with a number of cancer-related outcomes including prolonged survival, reduced fatigue, and enhanced surgical outcomes (46); however, its use within the exercise cardio-oncology field is limited. Here we describe the current evidence assessing the effect resistance exercise has on anthracycline-induced cardiotoxicities.

#### Murine Models

Cardioprotective effects have been observed in preclinical resistance training protocols implemented during anthracycline treatment. In a study by Pfannenstiel and Hayward (87) with a 12-week prehabilitation resistance-based intervention in rats receiving either DOX or saline infusion, resistance training was demonstrated to be cardioprotective against DOX-induced cardiac dysfunction. While there was still a drug effect in the DOX compared to the saline groups, the DOX-resistance group had significantly faster mitral and aortic blood flow velocities, higher fractional shortening, and higher systolic and diastolic function compared to the DOX-sedentary group. The authors proposed that this attenuation of cardiac function in the DOX-resistance trained group was a result of preserved myosin heavy chain isoform distribution and decreased oxidative stress (87). Similarly, Feitosa, Carvalho (86) found that 8-weeks of resistance training prevented a DOX-induced increase in diastolic arterial pressure and HR compared to the sedentary group receiving DOX, yet, both DOX groups had a significant treatment-induced reduction in systolic arterial pressure when compared to the placebo-sedentary group. Resistance training also prevented DOX-induced changes in spontaneous baroreflex sensitivity, sympathetic tone, vagal tone, left ventricular developed pressure, and oxidative stress. The authors proposed that the prevention of DOX-induced cardiac-related changes exhibited by the resistance exercise group may be associated with hemodynamic adjustments triggered by baroreflex sensitivity, cardiac autonomic tone, improved contractility, and reduced oxidative stress (86). Although these findings suggest that resistance-based exercise protocols may have cardioprotective effects, more studies are needed to provide further evidence especially using tumor-bearing rats receiving anthracyclines.

#### Clinical Interventions

To date, only one clinical trial examined the effects of resistance exercise on cardiotoxicity outcomes in cancer patients receiving anthracyclines. In the BEATE RCT, Schmidt et al. assessed a 12-week resistance training intervention in stage I-III breast cancer survivors (*n* = 101), of which 89.5% were receiving adjuvant anthracycline treatment (90). While resistance exercise was deemed feasible during adjuvant chemotherapy with a 71% adherence rate and exerted significant improvements in physical fitness, assessed by isokinetic and isometric muscle strength, role function and social function quality of life outcomes, and fatigue, no improvements in cardiorespiratory fitness were reported (90). Given the desirable effect resistance exercise has on cardiovascular health-related outcomes in the non-cancer population (108), and the previously described cardioprotective benefits identified in preclinical models receiving anthracyclines (86, 87), the unique cardiac-related effects of resistance exercise are worth further exploring within clinical trials among survivors who will, are, or have received anthracyclines.

### Multi-modal Exercise Interventions

Both aerobic and resistance exercise have independent, beneficial effects on cardiovascular-related outcomes that potentially lead to different improved effects on anthracycline-induced cardiotoxicities. Within the cancer exercise guidelines recommended by ACSM (46), combined aerobic and resistance exercise are encouraged so that their respective benefits are collectively obtained. Here we discuss the available clinical studies that utilize multi-modal exercise interventions, including combined aerobic and resistance exercise, to target anthracycline-induced cardiotoxicities. Currently, no preclinical studies have examined multi-modal exercise interventions.

#### Clinical Interventions

Kirkham, Virani (92) completed a non-RCT assessing the effects of an 8–12 week progressive combined aerobic and resistance exercise intervention on cardiac function among breast cancer survivors receiving anthracyclines (*n* = 37). Across the intervention, systemic vascular resistance significantly decreased compared to baseline in both the exercise and control groups, however, the decrease in systemic vascular resistance was significantly attenuated in the exercise group (−264 ± 482 vs. −444 ± 376 dynes·sec·cm^−5^), where the reduction in systemic vascular resistance in the exercise group was in response to an increase in vessel lumen radius. Cardiac output was also preserved in the exercise group compared to a significant increase from baseline in the control group. In contrast to the preservation of systemic vascular resistance and cardiac output, the exercise group had both a clinically and statistically significant decrease in VO_2peak_ (−2.6 ± 2.2 mL/kg/min), where a >1 mL/kg/min decline has been associated with 9–10% increased cardiac-related mortality risk (109, 110). However, the usual care control group did not have VO_2peak_ data, therefore, it is unclear if exercise attenuated the chemotherapy-induced reduction in VO_2peak_, or if chemotherapy blunts exercise training adaptations. In spite of this, a secondary analysis identified 61% of the cohort as low-adherers (defined by attendance ≤ 67% of sessions), who had a significant within group reduction in VO_2peak_, but this was only trending as different compared to the high adherers (*p* = 0.09). Of the sessions attended, the program was well adhered to as exhibited by 86% of participants reaching the intended aerobic intensity of 50–75% age-predicted HR reserve, and 96% of participants completed the intended aerobic duration of 20–30 min, therefore, highlighting this prescription as feasible during chemotherapy for breast cancer survivors. However, the results in this study should be interpreted with caution given the potential selection bias of the assessed cohort. This study compared the exercise group to a concurrent usual care control group from a separate study, previously described (63); survivors were given the choice to participate in the single group intervention exercise study, described here (92), or the RCT examining a single bout of exercise prior to the first anthracycline infusion compared to usual care (63).

During treatment, survivors may experience acute cardiotoxicities such as tachycardia and diastolic hypotension, which can result in dizziness, lightheadedness, and difficulty in changing body position, as such, clinicians should be aware of the pattern in which these cardiotoxicities occur and provide the necessary intervention to ensure improved survivor well-being (93). One such adjuvant strategy is the inclusion of exercise during treatment; Kirkham, Lloyd (93) examined the effect of a combined aerobic and resistance-based exercise intervention during treatment, that continued for 10 weeks post-treatment, on resting HR and blood pressure in breast cancer survivors stage I-III (*n* = 73). Treatment regimens placing survivors at high risk of cardiotoxic side effects, including anthracyclines, which 71% of the cohort were receiving, were significant predictors of a 5–8 beat worsening of resting HR and HR recovery during treatment. However, a high attendance of exercise sessions (≥67%) was associated with a 6-beat improvement in HR and HR recovery suggesting exercise to have a potential counteractive effect. Low aerobic fitness was additionally a strong independent predictor of elevated resting HR and impaired HR recovery both during and after treatment. This study continues to demonstrate the feasibility of exercise during treatment and provides critical insight into the prevalence of acute cardiotoxicities. While exercise is generally safe to complete when these acute cardiotoxicities are present, assessment precautions should be taken before exercise is begun as described within the safety measures of this study, which were taken from the ACSM contraindications to exercise for cancer survivors; if resting HR > 100 bpm, systolic blood pressure > 145 mmHg, or diastolic blood pressure > 95 mmHg, exercise should not be completed (46).

In a non-RCT, both Howden, Bigaran (57) and Foulkes, Howden (94) examined the same cohort primarily assessing the effect of exercise on VO_2peak_ and cardiac function after an 8–12 week intervention. In order to determine if the impact of exercise persists into survivorship, Foulkes, Howden (94) reported on the long-term effects, 12 months following anthracycline cessation, and Howden, Bigaran (57) reported on the effects of exercise during treatment. The exercise intervention consisted of a periodized aerobic interval training and resistance exercise intervention in breast cancer survivors receiving anthracyclines (*n* = 28) (57). As seen in Kirkham, Virani (92), exercise during treatment does not necessarily improve but does attenuate a decline in cardiorespiratory fitness, which is again demonstrated in this study where the usual care control experienced a 15% decline in VO_2peak_ that was significantly different to the 4% decline noted in the exercise group (*p* = 0.01). However, the exercise group had a significantly greater baseline VO_2peak_ and it is unclear if this difference was accounted for in the group x time comparison. Furthermore, anthracyclines induced a significant decline in resting measures of LVEF and hemoglobin, and an increase in troponin I (*p* < 0.001), which were not attenuated by exercise. However, Howden, Bigaran (57) did demonstrate that exercise measures of cardiac function better predicted cardiac impairment than resting measures and proposed the inclusion of exercise tests within the clinical setting. At 12 months following anthracycline cessation, Foulkes, Howden (94) (*n* = 17) reported a continued significant decline in VO_2peak_ for both groups with no between-group differences. Therefore, survivors who undertook exercise during chemotherapy were not protected from the acute or long-term chemotherapy-induced declines in VO_2peak_. However, exercise was not assessed during the 12 months post-treatment, making it unclear whether this change in functional capacity was treatment-related or if a detraining effect took place. Regardless, continued exercise post-treatment should be incorporated into survivorship care to prevent continued functional decline (Figure 3).

Mijwel, Backman (95) and Ansund, Mijwel (91) compared different combinations of multi-modal exercise programs. OptiTrain is a RCT conducted in breast cancer survivors (*n* = 206) receiving adjuvant chemotherapy including taxanes, anthracyclines, or a combination of the two, where survivors were randomized to one of three groups: resistance exercise plus HIIT, aerobic exercise plus HIIT, or usual care control (95). Although cardiovascular outcomes were not a focus of this study, VO_2peak_ (L/min) was measured, and in survivors not receiving taxanes, both the resistance plus HIIT and aerobic plus HIIT groups maintained VO_2peak_, whereas the usual care group had a significant decline. However, when the groups were compared, only the aerobic plus HIIT group was significantly different from usual care controls among non-taxane survivors. Nevertheless, the within-group maintenance of VO_2peak_ in the resistance plus HIIT in those not receiving taxanes, and the significant difference in VO_2peak_ among those who were receiving taxanes in the resistance plus HIIT group compared to usual care, demonstrates that as little as 9 min of HIIT twice a week in conjunction with resistance training may be a time-efficient way to gain the same preservation of cardiorespiratory fitness compared to high volume aerobic exercise plus HIIT.

Ansund, Mijwel (91) conducted a one-year follow-up of the OptiTrain trial. This study primarily assessed acute and chronic chemotherapy-induced cardiotoxicities monitored by cardiorespiratory fitness, cardiac troponin T, a biomarker used in detecting acute cardiac damage, and NT-pro-BNP, a protein secreted by cardiomyocytes in response to cardiac wall stress a marker of long-term cardiac remodeling (91). This follow-up study examined 88 survivors, of which more than 93% of each group received anthracycline treatment. During the 16-week exercise intervention, where survivors were concurrently receiving chemotherapy, cardiac troponin T significantly increased in all groups, suggesting this was treatment-induced, and values returned to baseline concentration at one-year follow-up. Nt-pro-BNP levels showed no significant difference between groups at baseline or 16 weeks, however, at the one-year follow-up, the usual care group was significantly higher than both exercise groups (91). Survivors who met the biomarker criteria for risk of decreased cardiac function (cardiac troponin T > 10 ng/ml at post-intervention and Nt-pro-BNP >100 ng/ml at 1 year follow up), most likely in the usual care control group, had significant declines in VO_2peak_ 2-years post-follow up compared to those who did not meet the criteria, suggesting an early change in these biomarkers may indicate compromised cardiovascular function. Further evidence is required into the usefulness of these biomarkers and their potential at identifying the most at-risk survivors which may then allow for the implementation of early intervention strategies.

While supervised in-clinic exercise often induces a superior physiologic benefit, given the face-to-face nature of the intervention and the real-time tactile adjustments that can be made, this form of exercise is not always feasible or desired. Smith, Ness (97) utilized a 12-week home-based combined aerobic and resistance intervention where weekly phone calls were completed to address any concerns and monitor exercise adherence. This study was a case series examining five survivors of childhood cancer (>10 years) diagnosed with subclinical anthracycline-induced cardiomyopathy that primarily assessed their response to exercise. No adverse events were noted, and compliance to exercise prescription was 86%, suggesting the feasibility and safety of home-based exercise in this population. Additionally, survivors showed improvements in VO_2peak_ (10.6%), ejection fraction (12.6%), and oxygen pulse (13.6%), while maximal HR response and respiratory exchange ratio were preserved. Järvelä and colleagues (96, 98) also examined the effect of home-based exercise (16 weeks) implemented post-treatment using a single group study design in childhood survivors of acute lymphoblastic leukemia (*n* = 17, *n* = 21, in the respective studies). Cardiorespiratory fitness and myocardial function were primarily assessed and significant improvements were observed for VO_2peak_, early diastolic mitral inflow velocity, and lateral early diastolic mitral annulus velocity, which are all useful measures in predicting cardiac events (111). Further studies with a comparison group are needed to confirm the benefits of home-based exercise and the effect it has on acute and long-term anthracycline-induced cardiotoxicities.

## Ongoing Trials and Current Research Gaps

Exercise cardio-oncology is an evolving field, thus, here we describe ongoing trials registered on ClinicalTrials.gov and NIH RePORTER, or published as a protocol paper, that assess survivors receiving anthracyclines, and its accompanying cardiotoxicities, within the context of exercise (Table 3). To evaluate research gaps within the field, we discuss the ongoing trials within the following categorizations that require further clarity: prescription of exercise, timing, study size and duration, and participant characteristics.

**Table 3:** Ongoing clinical trials examining the impacts of exercise on anthracycline-induced cardiotoxicities in cancer patients and survivors (as of September 2021).

**Principal investigator**	**Study design**	**Outcomes of interest**	**Population**	**Intervention groups**	**Intervention**
Hundley (112)	RCT	*Primary*: Attenuate physical inactivity, fatigue, exercise capacity, cardiac and cognitive function, strength, and HRQOL	Lymphoma, anthracycline-based chemotherapy	Individually tailored physical activity intervention	Not specified
Hundley (113)	RCT	*Parent trial aims:* *Primary*: 1. Design an automated MRI hardware/software platform for measuring and reporting LVF. 2. Changes in our MRI platform generated measures of LV volumes, EF, strain, myocardial T1 mapping, and aortic pulse wave velocity to predict pre- to 24-month post-anthracycline chemotherapy treatment differences in these same parameters. *Sub-trial aims:* *Primary*: 1. Feasibility of screening, enrolling, and randomizing patients. 2. Identification of barriers for participating in or adhering to the Physical Activity Intervention and Control Group. *Secondary*: Change in peak exercise cardiac output, A-V O_2_, VO_2_, LVF, cognitive function, HRQOL, 6MWT, fatigue	21 Non or Hodgkin lymphoma and stage I-IV breast cancer (18–85 years) On-treatment (350 mg/m^2^ of anthracycline therapy or combination of anthracycline (250 mg/m^2^) and subsequent paclitaxel or herceptin	Physical activity intervention; Healthy living instruction group (Control Group)	6 months Supervised: 1–2 x/week Home-based: 1–2 x/week Combined aerobic and resistance exercise
Grandy (114)	Single group	*Primary*: Feasibility (rate of recruitment) and adverse events associated with exercise program *Secondary*: Program adherence, attrition, cardiac function, cardiac disease risk, aerobic fitness, fatigue, QOL, and other cardiac rehabilitation measures	12 adult cancer patients on anthracycline chemotherapy (minimum dose of 100 mg/m^2^ of DOX or 120 mg/m^2^ of DAN or 150 mg/m^2^ EPI)	Moderate-intensity aerobic exercise	12-week Moderate-intensity aerobic exercise: Supervised group sessions, 2 sessions/week, 45 min/session aerobic with an additional warm-up and cool-down, intensity at 40–60% HRR
Grandy and Keats (115)	RCT	*Primary:* Change in LVF *Secondary:* Change in cardiac electric activity, aerobic fitness, blood biomarkers, QOL, and fatigue	100 breast cancer patients receiving anthracycline (minimum dose of 240 mg/m^2^ of DOX or 300 mg/m^2^ of DAN)	Exercise + standard care; Standard care	12-week Aerobic exercise: Home based, 2 sessions/week, 20–45 min/session based on intensity; intensity varying between low (35–45% heart rate) low-moderate (46–55%), high-moderate (56–70%), and high (71–85%).
Antunes et al. (116)		*Primary:* Change in cardiac (dsy)function, LV strain, resting LVF *Secondary:* Change in anthropometric measures, physical function, physical activity, HRQOL, fatigue	90 breast cancer stages patients receiving (neo)adjuvant anthracycline	Combined aerobic and resistance; Standard care	2-month Supervised group sessions; 3 sessions/week Aerobic exercise: 70 min/session (60 min of aerobic exercise), progressive volume and intensity based on RPE-scale with goal to reach intensity between 65–80% of heart rate reserve Resistance exercise: Upper body and lower body weight training, progressive volume and intensity using RPE-scale
Monzonís et al. (117)	RCT	*Primary:* Improve cardiac remodeling and enhance global cardiovascular risk profile *Secondary:* Change in functional capacity, 6MWT, VO2, HRQOL, chemotherapy tolerance, pain, disability, anthropometrics, biomarkers, dietary pattern, physical activity, lymphedema	122 breast cancer patients; post-treatment; treated with anthracyclines and / or anti-HER-2 antibodies (trastuzumab and / or pertuzumab)	Cardiac rehabilitation; Conventional management	Cardiac rehabilitation: Supervised exercise training (planned to be center-based, shifted to telematic due to COVID-19);
Christou (118)	RCT	*Primary:* 1. The effect all-extremity non-weight bearing exercise intervention, treadmill, and usual care on endothelial cell function and cancer therapy-related cardiac dysfunction. 2. Feasibility, tolerability and safety of all-extremity non-weight bearing exercise intervention compared with TE	68 breast cancer patients receiving (neo)adjuvant anthracycline, alkylating agent and/or taxane	All-extremity non-weight bearing; Treadmill aerobic exercise; Usual care	12-week Supervised; progress to 70% peak heart rate, 50 min/session, 3 days/week
Thavendiranathan and Adams (119)	RCT	*Primary:* Change in cardiorespiratory fitness *Secondary:* Change in cardiovascular disease risk factors, cardiovascular biomarkers, cardiac function, blood biomarkers, insulin sensitivity, BMI, physical activity levels, psychosocial health, QOL	696 young adult cancer survivors (18-39 years); post -treatment	Cardio-oncology rehabilitation (CORE); Standard care	6-month Aerobic exercise: HIIT training 2 times/week (1 supervised session and 1 home-based session), behavioral support

*HRQOL, health-related quality of life; RCT, randomized clinical trial; MRI, magnetic resonance imaging; LVF, left ventricular function; EF, ejection fraction; A-V O2, arteriovenous oxygen difference; VO_2_, oxygen consumption; 6MWT, 6-minute walk test; QOL, quality of life; DOX, doxorubicin; DAN, daunorubicin; EPI, epirubicin; HRR, heart rate reserve; ROM, range of motion; RPE, rating of perceived exertion; TE, treadmill exercise; UC, usual care; BMI, body mass index; HIIT, high-intensity interval training*.

### Prescription of Exercise

Previous anthracycline-based studies have considerable heterogeneity in the modes of exercise, and how they are prescribed e.g., frequency, intensity, duration, which continues to be present among ongoing trials. CORE, as previously described, has provided guidelines for the cardiac rehabilitation infrastructure required to address the unique exposures survivors at high risk of cardiotoxicities experience; however, these guidelines are yet to be evaluated in a clinical setting (48). In an ongoing trial by Thavendiranathan and Adams (119), cardiorespiratory fitness (VO_2peak_), in addition to other measures of cardiac function and health, is being compared between a CORE-based intervention group and standard of care control group over 24-weeks in survivors of pediatric, adolescent, and young adult cancers (diagnosed ≤ 39 years) who had received treatments known to increased CVD risk, including anthracyclines, and are now adults (≥ 18 years old). The results of this trial will provide insight into the effectiveness of the proposed CORE guidelines and how the guidelines may need to be amended to ensure survivors at risk of cardiotoxicities are receiving the most effective exercise prescription.

While heterogeneity of exercise modes and how they are prescribed, may be considered a limitation when trying to compare studies and assess the general effect of exercise on cardiotoxicities, it may also be considered a strength of the study in that it establishes the feasibility of different exercise protocols and their effect on cardiotoxicities for survivors on anthracyclines. For example, Christou (118) is comparing the tolerability of a 12-week novel all-extremity based exercise (mode unspecified) to treadmill walking and usual care to assess the effect on endothelial function in breast cancer survivors receiving chemotherapy, including anthracyclines. However, much of the published literature (57, 63, 92, 94, 96–98) and ongoing trials (113–116) focus on traditional aerobic- and resistance-based exercise prescriptions, as such, there is a lack of understanding as to the effect of other non-traditional modes such as HIIT, yoga, and circuit training. Of the collated list of ongoing trials, only one is prescribing a non-traditional mode of exercise, HIIT (119); however, Monzonís, Peña Gil (117) had to change the environment in which they supervised exercise due to COVID-19 and employed the use of virtually supervised exercise, a non-traditional setting of providing exercise. Further evaluation of the different modes, and the frequencies, intensities, and duration in which they may be prescribed referred to as the FITT (frequency, intensity, time, and type) principle of exercise prescription, will provide further understanding of the most effective prescription and the minimum thresholds in which benefits occur.

### Timing

For the majority of cancer survivors, some exercise is preferred over sedentary behavior, and it is never too late to begin participation in exercise; however, the time point that exercise is most effective within the cancer care continuum is an ongoing debate and is likely cancer, treatment, and survivor specific. As described in the previous section, the published literature spans the entire cancer care continuum with exercise interventions being implemented before, during, and after chemotherapy, and have all demonstrated improvement or preservation in one or more cardiac-related outcomes (Table 2). Therefore, exercising at any stage of the cancer care continuum for survivors prescribed anthracyclines will likely be beneficial. Nevertheless, the extent of its benefit in the long term may be dictated by when exercise is initially implemented. This research question is best answered through multi-decade follow-up studies, similar to that being done in the GAP4 (120) and CHALLENGE (121) trials, which assess the long-term impact of exercise on survival outcomes. On a smaller scale, clinical trials are still needed to determine the immediate benefits of exercise at the various stages of the cancer care continuum. Among the ongoing trials described within this review, none are targeting the use of exercise in the prehabilitation stage, six are examining the effect of exercise during chemotherapy (113–116, 118, 122), and two are examining the post-treatment/survivorship stage (117, 119). By understanding the optimal timing of exercise interventions, the cardioprotective benefits of exercise can be maximized and anthracycline-induced cardiotoxicities can be attenuated to a greater degree with integration into clinical practice or survivorship care models. Future studies are warranted to conduct adequately powered RCTs comparing exercise interventions administered prior to, during, and following anthracycline treatment with long-term follow-up for the understanding of sustainability of benefits.

### Study Size and Duration

With exercise being consistently demonstrated as a safe and feasible intervention among many cancer types and treatment regimens using small pilot studies and RCTs, larger multi-center trials are now needed to further emphasize the efficacy of exercise. While not an exercise intervention, Hundley (122) is conducting a 2-year prospective study to primarily assess the change in MRI measures of cardiovascular function, exercise tolerance through maximal and submaximal exercise capacity tests, and fatigue in breast cancer survivors on both anthracycline and non-anthracycline chemotherapies with comparison to age-matched non-cancer controls. This observational study has been actively recruiting since 2017 and has reported 403 enrollments as of September 2021. Large prospective cohort studies such as this one, can provide valuable information regarding what outcomes of interest change, the degree to which they change, and when these changes occur. This information can then be used to assess correlations among outcomes, which may assist in identifying who is at increased risk of poor cardiac health in the long term. Additionally, characterization studies like this can help decipher when the appropriate time is to intervene based on the timeline of outcome decline. Moreover, Thavendiranathan and Adams (119), previously described, are conducting a large 2- year exercise intervention trial examining VO_2peak_ with an estimated enrollment of 696 participants. The results of both these studies will be critical in understanding the trajectory of survivor health and the impact of exercise while on anthracycline treatment. However, both of these studies only follow survivors for 2 years and many of the debilitating cardiotoxicities are likely to develop years, if not decades, beyond treatment cessation. For example, survivors of childhood cancer have been reported to experience cardiotoxicities >40 years post-treatment (123), where those who are more active throughout the years post-treatment are less likely to experience cardiovascular events (22). Large, multi-decade databases such as the Childhood Cancer Survivors Study (124), Nurse's Health Study (125), and Women's Health initiative (126) are valuable resources in assessing the long-term effects of cancer treatment, and as the databases continue to expand, they will become essential in our understanding of anthracyclines and the impact exercise can have on cardiotoxicities.

### Participant Characteristics

There is a lack of clinical trials focusing on vulnerable cancer groups undergoing anthracyclines, including cancer types, survivors with a higher risk of cardiotoxicities, and racial minorities. Based on our review of the literature, the majority of studies are preclinical murine models with no specific cancer type or are clinical trials focused on breast cancer populations comprised of non-Hispanic Whites. As such, more studies covering a greater range of cancer types is required e.g., acute lymphoblastic leukemia, Hodgkin's and non-Hodgkin's lymphoma, and bladder cancers. In addition to expanding our understanding of the effect of exercise on anthracycline-induced cardiotoxicities across a number of different cancers, it is important to identify who the most vulnerable survivors are that would benefit the most from early intervention. For example, Hundley (113) is assessing the change in cardiovascular MRI measures taken prior to the initiation of anthracycline or other cardiotoxic cancer therapies, and 3 months later, to assess if this predicts cardiovascular-related outcomes at 24 months in Hodgkin's and non-Hodgkin's lymphoma and stage I-IV breast cancer survivors (Table 3). A smaller component of this parent trial is to assess the feasibility of combined aerobic and resistance-based exercise compared to a healthy living education group where the cardiac MRIs from the individuals in this pilot study will be compared to the survivors in the parent study. This trial is unique in that it may provide key outcomes worth measuring in the early stages of a cancer diagnosis which could help identify the most vulnerable survivors. Additionally, the same group has proposed a trial to assess how a physical activity intervention attenuates physical inactivity and preserves exercise capacity, cardiovascular and cognitive function, strength, and health-related quality of life for those with lymphoma (112). This trial also has a novel component where they will examine a newly developed magnetic resonance cardiopulmonary exercise treadmill testing method to measure cardiac function and peripheral factors to further understand the mechanics of how physical activity helps preserve exercise capacity and reduce fatigue (112). Vulnerable groups that should be further explored due to the already identified risk of developing anthracycline-induced cardiotoxicities include survivors who receive high doses of anthracyclines, elderly and pediatric cancer populations, and survivors with comorbid disease (e.g., hypertension and diabetes) or with pre-existing CVD. Finally, racial/ethnic minorities are also in need of further study as they are disproportionately underrepresented in exercise oncology, exercise-cardio-oncology, and anthracycline settings, and often have a higher risk of cardiac-related events due to pre-existing conditions and poor lifestyles (127, 128). To establish exercise as a standard of care strategy in reducing anthracycline-induced cardiotoxicities, further clinical trials involving these rarely studied and vulnerable groups are needed to confirm the translation of the benefits of exercise interventions to populations with other cancer types and characteristics.

Based on the current evidence, exercise is a viable non-pharmacological strategy that can be used to mitigate anthracycline-induced cardiotoxicities among cancer survivors. While the optimal prescription and timing of exercise to elicit the greatest benefit are still unknown, the discussed studies demonstrate exercise induces positive effects on cardiac-related outcomes throughout the cancer care continuum. Within our critical analysis of the published and ongoing studies in exercise cardio-oncology, we established a number of gaps within the field that should be addressed in future studies (Table 4). The next section discusses guidelines and considerations for the employment of exercise for cancer survivors on anthracyclines. This information will be useful for researchers and clinicians in developing future research protocols and in-clinic programs to further disseminate the value and clinical application of exercise for this vulnerable population.

**Table 4:** Research questions and considerations for future studies.

**Unanswered future research questions**	**Considerations**
What type(s) of exercise is(are) effective to address anthracycline-induced cardiotoxicity?	Modality of exercise (aerobic, resistance, or combined); cancer type; treatments.
What is the ideal time during the cancer continuum to intervene?	Pre-treatment, during treatment, or following treatment.
What is the optimal duration of an exercise intervention?	Timing of intervention, duration of exercise sessions, length of intervention, age of participant, diagnosis, presence of comorbid conditions.
Large, multi-center studies to better understand the feasibility and effectiveness of exercise to address anthracycline-induced cardiotoxicities.	Timing of intervention in treatment timeline, resources, setting of intervention (in-clinic/supervised, home-based/unsupervised, virtual/supervised, hybrid), availability of resources.

## Exercise Cardio-Oncology in Practice: Considerations for Implementation

### Should the Current Exercise Guidelines Be Challenged?

Exercise has consistently been deemed safe, feasible, and effective for cancer survivors before, during, and post-treatment, as such, ACSM developed generic exercise guidelines for cancer survivors, which have been previously described and are summarized in Table 1. While these are evidence-based guidelines, and the prescription variables have been identified as the most effective for cancer survivors, exercise should be individualized for each survivor, cancer type, and treatment. Therefore, clinicians and exercise specialists working with survivors with cardiotoxicities or aiming to prevent cardiac-related events, should also consider cardiology and cardio-oncology guidelines to address the unique needs of these survivors, and are also summarized in Table 1 (48, 129, 130). Before survivors can be referred to or prescribed an exercise program, special considerations must be given to the type of cancer, stage of the disease, survivor health, timing of treatment, treatment tolerance, and what the goal of exercise is (e.g., to improve cardiorespiratory fitness muscle strength) (47, 48, 129, 131). These factors can impact the duration, frequency, intensity, and modalities of exercise that are safe and effective for a survivor. Therefore, to support the integration of safely practiced exercise, the CORE guidelines emphasize the importance of individually tailoring exercise prescriptions, which will be essential for improved adherence to health behaviors and long-term cardiotoxicity outcomes in cancer survivors receiving anthracyclines (47, 48, 129, 131).

### Implementation of Exercise Cardio-Oncology in Clinical Practice

The setting in which exercise is performed e.g., supervised, self-directed, home-based, clinic-based, will have critical impacts on the accessibility and longevity for integrating exercise programs into cancer care settings. The CORE guidelines recommend the use of both supervised and self-directed exercise, depending on the needs of the survivor, due to a number of barriers when exclusively using supervised exercise, particularly in a clinic-based environment (48). Supervised, in-clinic settings provide a safe exercise environment for cancer survivors in that exercise physiologists can make real-time, tactile adjustments to ensure the correct execution of exercises. Additionally, survivors can exercise one-on-one with an exercise physiologist or in a supervised group setting that provides social support and accountability in adhering to exercise sessions. However, in-clinic sessions have a number of barriers relating to survivor adherence to the exercise intervention and may deter the development of long-term exercise habits as the burdens of commuting, number of other medical-related appointments, accessibility, cost, and sustainability are more pronounced than exercise interventions offered in a self-directed or virtually supervised home-based setting (48). Additionally, it is important to acknowledge the increased levels of stress and fatigue that a cancer diagnosis and treatment may have on survivors. Therefore, offering a hybrid or choice of in-clinic and home-based supervised exercise sessions may result in greater adherence to exercise programs. Self-directed exercise may be used to complement supervised sessions to increase the volume of exercise without restricting the survivor to specific appointment times. Self-directed exercise may also be prescribed if the barriers identified with supervised exercise are of concern; in this case, survivors may benefit from a one-off supervised exercise session, or a telephone/in-person consultation with an exercise physiologist to establish exercise goals and routine. However, self-directed exercise should only be recommended if the survivor is able to safely perform exercise without assistance and has clinician clearance. To further understand how exercise may be prescribed for survivors receiving anthracyclines to prevent, manage, or improve cardiotoxicities, the CORE guidelines recommend that adherence and feasibility of exercise interventions be incorporated in the design of interventions, which will allow for translation into realistic, clinical care settings (48). In the studies we reviewed, 10 utilized supervised/clinic-based sessions (16, 63, 88–93, 95, 103), three were self-directed and home-based (96–98), and two were combined supervised/clinic-based and self-directed/home-based sessions (57, 94). With the limited research on mitigating anthracycline-induced cardiotoxic outcomes with exercise-based interventions, future studies should prioritize targeted and realistic exercise programs that facilitate survivor adherence across the cancer care continuum, including supervised and self-directed settings and in-clinic, home-based, virtual, and commercial environments, where the latter two are yet to be examined.

### Exercise as Prehabilitation

There is a growing interest to utilize exercise in prehabilitation settings early on in the cancer care continuum, as diagnosis provides a “teachable moment” where survivors are often more willing to make healthy changes to their lifestyle (132). Additionally, incorporating exercise between diagnosis and treatment may have protective clinical benefits as demonstrated by a number of interventions described in this review (15, 63, 67, 69, 83, 87), and improve the survivor's “starting point” where they begin treatment with a higher fitness level, improved body composition etc. as depicted in Figure 3. Furthermore, survivors may be in a healthier state prior to starting treatment and, therefore, are more likely to be able to complete a greater volume and/or higher intensity of exercise, which may lead to greater benefits and adherence, compared to starting an exercise program during or immediately post-treatment where survivors a likely in a weaker state. The use of exercise prior to anthracycline treatment is in alignment with the recent recommendations from the CORE guidelines for cancer populations at risk of cardiac-related outcomes (48). However, since the timing between cancer diagnosis and the start of chemotherapy treatment is relatively short (i.e. typically 1–12 weeks), future research is needed to understand how acute bouts of exercise immediately prior to anthracycline treatment may impact anthracycline-induced cardiotoxic outcomes (50, 133).

### Exercise During Treatment

Exercise during anthracycline treatment has been established as safe and beneficial for cancer survivors (48, 129, 130, 134–136). In the current review, we have addressed how exercise has been shown to be safe and effective for cancer survivors in both the neoadjuvant and adjuvant anthracycline settings, which is associated with improvements in the efficacy of treatment and reduces cardiotoxicity risk in survivors who exercise regularly (Table 2). Considerations that may need to be addressed when prescribing exercise while receiving anthracyclines includes what and when treatment-related side effects are experienced. Understanding the cycle of chemotherapy side effects e.g., which days after infusion survivors feel the best or worst, will assist in developing appropriate individualized exercise protocols. Furthermore, the number of medical-related appointments may also be of concern when prescribing exercise during this period, as it may dictate the preferred setting in which the survivor may prefer to exercise. For example, virtually supervised, as opposed to in-clinic exercise, may be more appealing given survivors will already be attending a number of chemotherapy infusion appointments. On the other hand, exercise bouts conducted at the hospital during an anthracycline infusion, or immediately before or after, may also provide further benefits including chemotherapy tolerance and effectiveness (63). However, exercise during the infusion is a relatively new concept and its feasibility and clinical application is yet to be established. In alignment with the current CORE and non-cancer cardiology guidelines, exercise needs to continue to be integrated into cancer treatment standard of care to support the health of cancer survivors at an increased risk for long-term cardiotoxic outcomes from anthracycline-treatment (48, 129).

### Exercise Post-Treatment

Long-term cardiotoxic side effects of anthracyclines can be present anywhere from 1 year to decades after treatment completion, therefore, exercise post-treatment should be considered for long-term health and wellbeing of cancer survivors to improve, manage, or prevent late occurring anthracycline-induced cardiotoxic outcomes. Similar to diagnosis, the period of survivorship is also considered a “teachable moment” as survivors have gone into remission and are often willing to make the necessary lifestyle changes to prevent long-term side effects and cancer recurrence (132, 137). During the survivorship period, cancer care providers should utilize this time to nurture health behavior change and minimize the long-term impacts of cancer and cancer treatment (132, 137–139). There is established research that already supports the use of exercise to reduce the risk of cancer-related co-morbidities and improve health benefits, quality of life, and cardiovascular-related outcomes for survivors of numerous cancers (19, 46–48, 140–142), however, exercise studies during survivorship that exclusively target those receiving anthracyclines are lacking. Given that anthracyclines elevate the risk of cardiotoxicity and cardiovascular morbidity and mortality among cancer survivors, the incorporation of exercise-based interventions into established local and national survivorship programs should be explored, including clinic-based or commercially available group-exercise programs and resources for supervised and self-directed exercise programs. For example, *LIVESTRONG* is an exercise program for cancer survivors available at YMCA facilities in the United States (143, 144). This program offers a 12-week, supervised, individually tailored, small group-based exercise program with two sessions per week at little to no cost to the survivor. In one study assessing the safety and effectiveness of the *LIVESTRONG* program in cancer survivors both on and off-treatment, researchers found improved levels of physical activity, fitness, quality of life, and cancer-related fatigue (144). A similar exercise program by Rajotte, Yi (145) also conducted among cancer survivors of various diagnoses and ages in local YMCA facilities, found significant improvements in blood pressure, strength, fitness, and quality of life (145). Although these programs are not widely available, the expansion of such programs can be utilized to benefit cancer survivors who had anthracyclines. LIVESTRONG, and similar clinic-based and commercially available programs (146), highlight the sustainability and feasibility of these programs in real-world settings. In support of the CORE guidelines, community-based programs may be more realistic and accessible to integrate into everyday life and offer benefits to cancer survivors treated with anthracyclines, while still allowing supervised, tailored exercise prescriptions that can best support vulnerable cancer populations. There is a need for more accessible and affordable exercise programs for cancer survivors to access within their community as part of survivorship to minimize the risk of long-term anthracycline-induced cardiotoxicity.

### Multidisciplinary Approaches

In order to have successful integration of exercise-based programs within cancer care, there is a need for multi-disciplinary teams in the prescription, delivery, and promotion of exercise across the cancer care continuum. Oncology is inherently multidisciplinary, as the collaboration across various healthcare professionals, community care resources, insurance companies, and referral pathways is imperative for comprehensive treatment and care (147). Standard of care teams are often cancer-type and treatment specific and may include specialists in medical oncology, radiation oncology, surgical oncology, cancer site specialists, primary care providers, nurses, physical therapists, and nutritionists (148). Exercise programs designed for sustained health benefits should be guided by an exercise physiologist or specialist, yet exercise specialists are not usually a part of the standard of care team (149, 150). The lack of appropriate exercise specialists on the standard of care teams creates a disconnect in the referral pathways for exercise-based programs in critical cancer populations. There is a clear gap between research into the benefits of exercise throughout the cancer care continuum and the incorporation of exercise into the standard of care. Elbourne, Soo (151) reported that fewer than 30% of clinicians working with prostate cancer survivors were aware of referral pathways for a supervised exercise program, and less than 16% of clinicians engaged in conversations with survivors about exercise (151). Though the integration of exercise as adjuvant medicine into the cancer treatment plan was low, nearly 73% of the clinicians in the study agreed that exercise counseling should be incorporated into cancer care (151). In a similar survey by the American Society of Clinical Oncology, 78.9% of oncology clinicians supported recommending physical activity to cancer survivors, yet less than 23% referred survivors to exercise programming (152). To best support the long-term health, wellbeing, and quality of life of cancer survivors prescribed anthracyclines, the standard of care teams should integrate exercise physiologists and rehabilitation specialists into the multidisciplinary teams to ensure that early referrals for exercise cardio-oncology programs can be incorporated (153–157).

### Referral Programs

There are no established best practices regarding referrals for survivors who are at high risk of anthracycline-induced cardiotoxic outcomes, as referrals for exercise are at the discretion of the survivor's physicians and standard of care team (48, 149, 156–158). To ensure referrals in cancer care settings, there needs to be an increased awareness of the importance of exercise as well as appropriate environments to refer to in order to implement exercise as a non-pharmacological strategy to mitigate cardiotoxic outcomes (48, 155–157). In a recent paper by Schmitz, Campbell (157) oncologists and providers within the standard of care team were called to actively “assess, advise, and refer” survivors for physical activity regularly throughout the cancer care continuum. However, one of the prominent barriers to successful utilization of referrals and implementation of exercise programs is the lack of oncologist and provider engagement in the referral process, which hinders the implementation and longevity of exercise programs for cancer survivors (147). For exercise referral to be effective, increased educational opportunities for health care providers and cancer care team members should be integrated into cancer-related conferences, online education modules, and healthcare professional development workshops. These continued education opportunities will aid in expanding awareness and advocacy for exercise in cancer standard of care, which will support the long-term health of at-risk survivors treated with anthracyclines to reduce cardiotoxic outcomes (48, 138, 149, 150, 157, 159).

Referrals for exercise should be unique for each survivor, as cancer type, treatment effects, health/medical status, current activity levels, ability level, and preferences for the type of exercise programming need to be accounted for (48, 157). To support clinicians in the process of assessing, advising, and referring survivors to exercise programs in the standard of care, ACSM developed the *Moving Through Cancer* initiative (160). *Moving Through Cancer* links clinicians with resources to connect survivors with established physical activity guidelines, as well as a directory with exercise professionals and programs in the community (160). Alongside increased education of exercise in the standard of care, the usage of referral programs like *Moving Through Cancer* that connect survivors with community-based exercise professionals and programs will greatly enhance the quality of exercise cardio-oncology care for cancer survivors at risk for anthracycline-induced cardiotoxic effects.

## Conclusion

The management of anthracycline-induced cardiotoxicities is a serious unmet clinical need. Current scientific evidence suggests that exercise cardio-oncology interventions may be effective non-pharmacological approaches to protect or reverse the cardiotoxicities from anthracyclines. Preclinical studies support the benefits of exercise through various biological mechanisms of anthracyclines in conjunction with physiological effects of exercise, from cellular signaling in cardiac cells to systemic adaptations in the cardiovascular system, while the mechanisms of action in clinical studies need to be elucidated. Furthermore, several clinical studies have focused on the effects of exercise throughout anthracycline trajectories and collectively support the use of exercise as a feasible and safe modality that can prevent treatment-induced changes in cardiorespiratory fitness, biomarkers associated with cardiac damage, and cardiac function. There has been a growing interest in this field, accompanying research funding opportunities specifically targeting cancer treatment-related cardiotoxicities such as the National Cancer Institute and the National Heart, Lung, and Blood Institute (PA-19-112). Further large, multi-center studies with long-term follow-ups are needed to provide comprehensive evidence considering different exercise modalities, timing, intensity, implementations, and dissemination, and more vulnerable and understudied subgroups of cancer survivors before, during, and after anthracyclines.

## Author Contributions

All authors listed have made a substantial, direct, and intellectual contribution to the work and approved it for publication.

## Conflict of Interest

The authors declare that the research was conducted in the absence of any commercial or financial relationships that could be construed as a potential conflict of interest.

## Publisher's Note

All claims expressed in this article are solely those of the authors and do not necessarily represent those of their affiliated organizations, or those of the publisher, the editors and the reviewers. Any product that may be evaluated in this article, or claim that may be made by its manufacturer, is not guaranteed or endorsed by the publisher.

## Abbreviations

ACS: American Cancer Society
ACSM: American College of Sports Medicine
AHA: American Heart Association
baFMD: brachial artery flow-mediated dilation
CORE: Cardio-Oncology Rehabilitation
VO_2peak_: cardiorespiratory fitness
CVD: cardiovascular disease
cIMT: carotid intima media thickness
DOX: doxorubicin
HR: heart rate
HIIT: high intensity interval training
LVD: left ventricular dysfunction
LVEF: left ventricular ejection fraction
RCT: randomized control trial
ROS: reactive oxygen species
Top2: topoisomerase II.
